# Cryptic female choice favours sperm from major histocompatibility complex-dissimilar males

**DOI:** 10.1098/rspb.2013.1296

**Published:** 2013-10-22

**Authors:** Hanne Løvlie, Mark A. F. Gillingham, Kirsty Worley, Tommaso Pizzari, David S. Richardson

**Affiliations:** 1 Department of Zoology, Edward Grey Institute, University of Oxford, Oxford OX1 3PS, UK; 2 Department of Zoology, Stockholm University, 10691 Stockholm, Sweden; 3 School of Biological Sciences, University of East Anglia, Norwich Research Park, Norwich, Norfolk NR4 7TJ, UK

**Keywords:** genetic relatedness, major histocompatibility complex, postcopulatory sexual selection, sperm choice

## Abstract

Cryptic female choice may enable polyandrous females to avoid inbreeding or bias offspring variability at key loci after mating. However, the role of these genetic benefits in cryptic female choice remains poorly understood. Female red junglefowl, *Gallus gallus*, bias sperm use in favour of unrelated males. Here, we experimentally investigate whether this bias is driven by relatedness *per se*, or by similarity at the major histocompatibility complex (MHC), genes central to vertebrate acquired immunity, where polymorphism is critical to an individual's ability to combat pathogens. Through experimentally controlled natural matings, we confirm that selection against related males' sperm occurs within the female reproductive tract but demonstrate that this is more accurately predicted by MHC similarity: controlling for relatedness *per se*, more sperm reached the eggs when partners were MHC-dissimilar. Importantly, this effect appeared largely owing to similarity at a single MHC locus (class I minor). Further, the effect of MHC similarity was lost following artificial insemination, suggesting that male phenotypic cues might be required for females to select sperm differentially. These results indicate that postmating mechanisms that reduce inbreeding may do so as a consequence of more specific strategies of cryptic female choice promoting MHC diversity in offspring.

## 1. Introduction

Offspring of genetically similar parents often suffer reduced fitness, either as a result of inbreeding depression [1,2] or reduced genetic variation at specific loci [3]. Loss of variation at key functional loci, such as those of the major histocompatibility complex (MHC), may be especially detrimental. The highly polymorphic MHC genes encode antigen-presenting molecules that are central to the vertebrate acquired immune response [4,5]. MHC class I genes are associated primarily with intracellular pathogens, whereas MHC class II genes interact with extracellular pathogens [6]. Reduced diversity at these MHC loci can therefore compromise an individual's ability to combat pathogens [7–10], and females should select partners to optimize the genetic diversity of their offspring [9–13]. However, female choice is often limited, for example because multiple males are able to coerce a female into mating [14,15]. When this happens, females might be able to bias fertilization in favour of genetically dissimilar males by using the ejaculates of individual partners differentially during or after copulation, a process known as cryptic female choice [16–18]. In principle, cryptic female choice might allow optimization of offspring MHC by biasing sperm use in response to the females' MHC similarity to a male [12,18–21]. However, this hypothesis remains little explored and empirically unresolved. Consistent with general expectations, some studies have found evidence of fertilization bias promoting the MHC heterozygosity of offspring, favouring males that are either MHC-dissimilar to the female [22,23] or those that are more MHC-heterozygous [24–26]. However, other studies have failed to detect similar effects [27] or found a bias for MHC-similar males [28,29]. Furthermore, cryptic female choice is notoriously difficult to study owing to difficulties in controlling for precopulatory mechanisms, disentangling male- from female-driven processes, and distinguishing between differential sperm use and differential zygote mortality as sources of paternity bias [30,31]. Importantly, because genetic relatedness and MHC similarity are often correlated [32,33], the relative importance of these factors in strategies of cryptic female choice has been particularly difficult to separate. Here, we address these challenges and experimentally investigate the relative roles of mate relatedness and mate similarity specifically at MHC loci in cryptic female choice in a population of red junglefowl, *Gallus gallus*.

The red junglefowl, the wild ancestor of the domestic chicken [34], offers an excellent opportunity to disentangle the role of MHC similarity and genome-wide relatedness in patterns of cryptic female choice. First, under the natural conditions in which social groups live, females are polyandrous and have limited precopulatory control of mate choice because the majority of copulations are forced on females by males [35,36]. However, females can retain some control of offspring paternity through cryptic female choice [37–39]. Second, the minimal size of the MHC of domestic chickens and red junglefowl, with the B-complex containing just two class I loci and two class II loci, may have intensified selection on these genes [40]. Clearer connections between variation at these MHC loci and immune traits or pathogen resistance have been made in the chicken than in virtually any other animal [41,42]. For example, reduced MHC diversity has been shown to increase susceptibility to pathogens in chickens [43,44] and to result in increased pathogen-induced mortality in the red junglefowl [10]. We would therefore predict females to benefit from exerting MHC-based cryptic female choice. Third, under natural conditions, limited dispersal by both sexes results in a significant risk of inbreeding [45,46]. Both males and females can discriminate kin [38], although, consistent with theory [14,47,48], inbreeding avoidance is weaker in males than in females. We have previously shown that, when presented with the opportunity to mate with a single female, males typically inseminate their own full-sibling sisters rather than avoiding inbreeding [38]. Females, on the other hand, appear to reduce the risk of inbreeding by selecting against the ejaculates of their brothers after mating [38]. The functional significance of this pattern of cryptic female choice remains unclear. One possibility is that females bias sperm use directly in response to genetic relatedness. However, an alternative hypothesis, suggested by the often strong relationship between relatedness and MHC similarity [32,33], is that cryptic female choice acts in response to MHC similarity, and inbreeding avoidance is an outcome of a more specific strategy based on MHC-based benefits.

In this study, we experimentally disentangle these fundamental mechanisms by taking advantage of an MHC-genotyped population of red junglefowl [49,50] in which genetic relatedness and MHC similarity are only weakly correlated (see Material and methods). We first confirm the previously reported female response to male relatedness following natural mating under experimental conditions. We then introduce information on MHC similarity between partners to test the extent to which this bias is explained by genetic relatedness *per se*, and/or by similarity specifically at the different MHC class I (BF1 and BF2) and class II (BLB1 and BLB2) loci. Within this analysis, we also assess the effect of the differentially expressed major and minor loci (BF2, BLB2 versus BF1, BLB1, respectively; major loci being the more dominantly expressed [44]). Finally, we explore whether patterns of cryptic female choice observed following natural mating are maintained following artificial insemination when females are experimentally prevented from gaining access to male phenotypic cues.

## 2. Material and methods

### (a) Study population

Experiments were conducted in January–February 2005 and March–April 2006 on a captive population of individually marked red junglefowl at the Swedish Agricultural University, Skara, Sweden (2005: *n*_females_ = 52, *n*_males_ = 45; 2006: *n*_females_ = 35, *n*_males_ = 27). Birds were kept indoors under constant conditions (12 L : 12 D cycle) and according to Swedish ethical legislations (Gothenburg Ethical committee, permission number 192–2004). Birds were admixed prior to the experiments, and the birds used were pedigree-bred for two generations (for further details, see [39]). Birds defined as ‘related’ were full-siblings in the pedigree, and thus had 0.5 probability of sharing a gene identical by descent in the past two generations (i.e. coefficient of relatedness, *r* = 0.5). ‘Unrelated’ birds were less related than half-cousins in the pedigree (*r* < 0.0625). An index of relatedness [51] based on allele similarity across 13 microsatellite loci confirmed patterns of pairwise relatedness (mean relatedness, *r*: 0.46 ± 0.22 versus −0.047 ± 0.056 for ‘related’ and ‘unrelated’ pairs, respectively).

Single-locus typing of both class I and class II MHC loci was undertaken using primers developed from domestic chicken [52,53] in combination with reference strand conformation analysis (for detailed methods, see [49,50]). This method is able to resolve all the sequences identified in this population of red junglefowl [49]. Compared with other vertebrates, the MHC of the fowl is simple and well understood [40,44], containing just two MHC class I loci (BF1 and BF2) and two MHC class II loci (BLB1 and BLB2) [40,54]. BF2 and BLB2 loci are termed ‘major loci’, whereas BF1 and BLB1 are termed ‘minor loci’ because the former are expressed 10 times more than the latter [44]. In the study population, there are nine class I alleles (six major and three minor) and 10 class II alleles (five major and six minor including the one found in both class II loci). For further details, see [10] (electronic supplementary material, figure S1). All alleles identified represent unique amino acid sequences [49]. MHC similarities between birds were calculated as 2*x*/*n*, where *x* is the number of alleles shared between a male and a female and ‘*n*’ is the total number of alleles present in the two birds. We calculated proportion of alleles shared by the male and female separately for (i) MHC class I major, (ii) MHC class II major, (iii) MHC class I minor and (iv) MHC class II minor loci, and (v) an index of overall MHC similarity (MHC alleles shared across all four loci). In our study, relatedness and MHC similarity between partnered birds were only weakly correlated (Spearman correlation coefficients between different measures of MHC similarity and relatedness ranged from *r*_s_ = 0.17 to 0.24, *n*_dyads_ = 53). MHC similarity between partnered birds, calculated at different MHC loci (e.g. similarity at class I minor versus class II major) were moderate to strongly correlated (Spearman, *r*_s_ = 0.61–0.83, *n*_dyads_ = 53, among the different MHC loci).

Birds were separated at hatching and randomly assigned into different groups visually isolated from each other (*n*_groups_ = 4, each with 12–18 individuals of mixed sex). Because prior social familiarity might influence kin recognition [55] and trigger inbreeding avoidance responses [38], we blocked for social familiarity; both males that mated with a given individual were either (i) socially familiar (i.e. raised together) or (ii) socially unfamiliar (i.e. not previously met).

Between ejaculation, males were physically isolated from females for at least 48 h to ensure replenishment of sperm supplies, whereas females were isolated from males for at least 10 days to ensure depletion of stored sperm [56]. Birds were sexually mature (more than eight months), and were 13–14 and 27–28 months old in 2005 and 2006, respectively, thus within their sexual prime [57]. Experiments were run blind with respect to genetic similarity and social familiarity between individual birds.

### (b) Controlled natural mating experiment

This experiment allowed us to test the effect of relatedness and MHC similarity between partners on the amount of sperm that reached the females' eggs while reducing female precopulatory mate choice bias by allowing only predetermined, staged matings to occur. One female at a time was presented by H.L. to a single male (thus reducing any potential ‘holder’ effects), facing the male for 1 min, after which the female was turned around and presented in a soliciting position for 20 min, or until the male copulated twice with the female [38,50,58]. Each female was behaviourally successfully copulated with, and inseminated by, a related and an unrelated male on occasions separated in time (a minimum of 10 days; *n*_females_ = 36, *n*_males_ = 30). Females were randomly assigned to copulate with a related or an unrelated male first. The egg laid on the first day after insemination was discarded as this is ovulated before sperm could have fertilized it [56,59]. For each egg laid over the following 10 days, we measured the number of hydrolysis points on the outer perivitelline layer (PVL) of the yolk caused by individual live sperm cells around the time of fertilization. In fowl, the probability that an ovum is fertilized is a function of the number of sperm trapped within the PVL [59]. Therefore, variation in the number of sperm-induced hydrolysis points on eggs laid over successive days following an insemination provides an accurate measure of the amount of sperm initially stored by a female, the rate at which sperm were released from the sperm storage tubules, and the probability of fertilization of individual eggs [59,60]. Furthermore, this measure is positively associated with number of sperm inseminated [59,60], and the probability that a given male fertilizes a female's eggs, also under sperm competition [61]. Being a continuous variable, this measure represents a more sensitive measure of the competitive performance of an ejaculate than binary data on the fertility or paternity of an egg. The number of sperm-induced hydrolysis points on four successive non-overlapping areas of the PVL centred around the blastodisc were counted using a Leitz Wetzlar Ortolux microscope with a Heine phase contrast condenser and 25× magnification, following an established protocol [38,58,62]. The highest numbers of hydrolysis points counted on eggs produced by a male–female dyad (‘highest sperm number on eggs’) were used for further analyses (see below). The analyses were restricted to the first three eggs that contained sperm because polyandry under natural conditions would mean that the fertilization window for a single copulation would only last for such a period of time [62]. Counts of hydrolysis points on PVL follow not only a logarithmic pattern of decline over time so the highest count is typically restricted to eggs laid in the first few days following insemination, but also providing an accurate approximation of sperm retention throughout the trial [38,62]. Assessing sperm use in this way, rather than calculating fertilization success of competing males by genotyping offspring, enabled us test for patterns of cryptic female choice without the confounding effect of any postzygotic patterns (e.g. inbred/MHC-homozygous embryos suffering higher mortality [30,63]). When the eggs of a female no longer had any hydrolysis points, the female was then inseminated by the opposite type of male (related or unrelated) and hydrolysis points were counted again.

All copulations were video recorded with Sony Hi8Xr TRV66E (2005) and Sony DCR VX-1000E (2006) cameras, focused on the female cloaca. An ejaculate was considered ‘accepted’ when either the ejaculate was observed entering the vagina through contractions of the female cloaca or no semen was observed exiting the female cloaca. An ejaculate was considered ‘ejected’ when semen was observed exiting the female cloaca following cloacal contact between the male and the female, following an established protocol of demonstrated repeatability [39].

### (c) Allocation trials

Because differential sperm allocation could potentially explain variation in the number of sperm found on eggs, a set of ‘allocation trials’ was performed to quantify sperm allocation from specific males to specific females. In a replicated set of matings, the focal males were allowed to copulate with the same female (as for the controlled natural mating experiment) on a separate mating occasion (minimum 10 days apart). During these copulations, females were fitted with harnesses preventing insemination and facilitating ejaculate collection [38,50,58]. Ejaculates were collected and measured to the nearest 1 µl with a Gilson pipette and sperm numbers were calculated according to the previous study [64]. The amount of sperm allocated by a male to a female (‘ejaculate sperm number’) during the allocation trials was included as a variable in the analyses of controlled natural mating for the same dyad (see ‘Statistical analyses’).

### (d) Artificial insemination experiment

To investigate whether precopulatory phenotypic cues could influence the number of sperm found on eggs, we conducted an experiment where ejaculates were inseminated artificially. A semen sample was obtained from a male by abdominal massage [56], homogenized and equal volumes inseminated approximately 2 cm into the vagina from the cloaca (prior to the sperm storage tubules) of two females; one being ‘related’ and one ‘unrelated’ to the male (*n*_females_ = 33, *n*_males_ = 21). The total volume obtained was inseminated, with volumes varying across females from 65 to 150 µl, which is within the range of ejaculate volume obtained in natural copulations in this study (mean ± s.e.: 120.9 ± 9.4 µl, median: 96.5 µl). No ejection of ejaculates after artificial insemination was observed. The number of sperm-induced hydrolysis points on the PVL of eggs, produced in the following 10 days, was counted as described for the ‘controlled natural mating’ experiment (see above).

### (e) Statistical analyses

#### (i) Genetic similarity

Only one measure of MHC similarity was entered in a statistical model at a time owing to moderate to high correlation between MHC measures (see ‘Study population’, above). But because relatedness was only weakly correlated with MHC similarity, both relatedness and one measure of MHC similarity were entered in the same model. We then compared the explanatory power of models, each including one of the five MHC measures.

#### (ii) Social familiarity

Because social familiarity between females and sperm donors was not correlated with relatedness or MHC similarity (Spearman, *r*_s_, range: 0.01–0.17, *n*_dyads_ = 53), and the ‘highest sperm number on eggs’ between females mated with unfamiliar or familiar males did not differ (Mann–Whitney *U*-test, *Z* = −0.27, *p* = 0.78), we pooled data from the trials conducted with familiar or unfamiliar birds for further analyses.

#### (iii) Female sperm retention

For the controlled natural mating we conducted three analyses, and for the artificial insemination experiments two analyses, investigating variation in female sperm retention using generalized linear mixed models (GLMM).

First, we investigated overall patterns of differential female sperm retention by analysing variation in ‘highest sperm number on eggs, all clutches’ including all clutches (i.e. with or without sperm; see below). We used a GLMM with a Poisson error distribution, ‘relatedness’ and ‘oviposition day’ (i.e. day 1–3 when the egg was laid), and MHC similarity as categorical effects. An additional covariate ‘ejaculate sperm number’ (i.e. the number of sperm inseminated by the same male to the same female, in ‘allocation trials’) was entered. ‘Female identity’ and ‘male identity’ were entered as random effects in the analyses.

We then conducted two separate analyses to further investigate patterns of female sperm retention and avoid any potential problem related to a zero-inflated Poisson distribution. We first analysed variation in the risk that the female failed to store any sperm from an insemination, as reflected by the presence or complete absence of sperm-induced PVL hydrolysis points on any of the eggs produced by a female during a trial (‘sperm absence’). Second, we restricted our analysis to trials that resulted in PVL hydrolysis points and analysed variation in sperm reaching the eggs by comparing the highest sperm number counted on an individual egg across trials. The effect of ‘relatedness’ and MHC similarity between partners on the likelihood of ‘sperm absence’ was analysed with a Binomial error distribution. Variation in the ‘highest sperm number on eggs’ had a Poisson error distribution. ‘Oviposition day’ was entered into models of ‘highest sperm number on eggs’ because the day with the highest sperm number differed slightly between individual females, but not into ‘sperm absence’ analyses because only one value was entered per female–male combination in these models. The models were otherwise built as described above. In the artificial insemination experiments, the total volume of each ejaculate was inseminated, thus sperm number could not be calculated. ‘Ejaculate volume’ was entered as a continuous covariate, instead of ‘ejaculate sperm number’ (as for the controlled natural mating experiment), in these analyses. However, to control for variation in ‘ejaculate volume’, analyses of variation in sperm use following artificial insemination were conducted by nesting ‘female identity’ in ‘male identity’.

The number of females and males varies in the analyses of the different responses and experiments depending on whether females laid eggs and whether these eggs contained sperm. In the controlled natural mating experiment, ‘highest sperm number on eggs, all clutches’ and ‘sperm absence’: *n*_clutches_ = 53, *n*_females_ = 30 and *n*_males_ = 25, for the ‘highest sperm number on eggs’: *n*_clutches_ = 33 (thus excluding clutches with no sperm, *n*_clutches_ = 20), *n*_females_ = 29, *n*_males_ = 22. In the artificial insemination experiment, ‘sperm absence’: *n*_clutches_ = 43, *n*_females_ = 30 and *n*_males_ = 21, ‘highest sperm number on eggs’: *n*_clutches_ = 33 (thus excluding clutches with no sperm, *n*_clutches_ = 10), *n*_females_ = 25 and *n*_males_ = 19.

Model selection is a common analytical approach used to choose models that best fit the data [65] and allows comparison of alternative models with correlated parameters that can cause problems with collinearity if included in a single model. We conducted model selection based on Akaike information criterion (AIC) values. AICc values (corrected for small sample sizes with greater penalty for extra parameters) and AIC weights (ω) were obtained using MuMIn in R, compared within each experiment and response variables analysed. Lower AICc values and higher *ω* values imply a better goodness of fit of models, and thus a better ability to explain variation in the data. Accepted convention is that models where the change in AICc compared with best-ranking model is less than 2 (*Δ*AICc < 2) are equivalent (and all such equivalent ‘best models’ are presented in the results below), whereas models with *Δ*AICc > 2 are less supported [65] (and thus not shown). AIC*ω* is used to assess the relative support for models, while the sum of AIC*ω* for each variable occurring in the supported models (**∑**AIC*ω*, obtained for all models with cumulative weight 0.95; electronic supplementary material, table S1) gives the relative importance of that variable [65].

#### (iv) Male differential sperm allocation

Variation in male sperm allocation (‘ejaculate sperm number’, transformed to obtain normality by subtracting the population mean and dividing by the population standard deviation) was investigated through separate GLMMs with Gaussian error distribution, entering ‘relatedness’, ‘social familiarity’ or one of the different measures of ‘MHC similarity’ as factorial effects, and including ‘male identity’ and ‘female identity’ as random effects.

#### (v) Female ejaculate ejection

Female ejaculate ejection was observed in 16 out of 37 mating trials. The probability of ‘female ejaculate ejection’ was investigated through separate GLMMs with Binomial error distribution, entering ‘relatedness’, ‘social familiarity’ or one of the measures of ‘MHC similarity’ as a factorial effect, or ‘ejaculate sperm number’ as a continuous effect, and including ‘male identity’ and ‘female identity’ as random effects.

All analyses were performed in R 2.10.1.

## 3. Results

**Figure 1.**
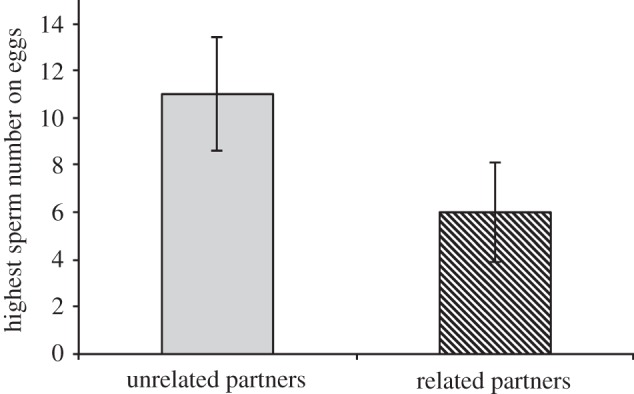
The relationship between extent of sperm retention in female red junglefowl and relatedness between partners, following insemination in the controlled natural mating experiment. Females retained more sperm (‘highest sperm number on eggs, all clutches’) following insemination by unrelated partners (grey column, ‘unrelated’ partners were less related than half-cousins in the pedigree) compared with insemination by related males (striped column, ‘related’ partners were full-siblings in the pedigree; table 1*a*). Data are presented as ±s.e.m. and include females that produced clutches both with and without sperm (*n*_clutches_ = 53, *n*_females_ = 30).

In the controlled natural mating experiment, females produced eggs with fewer sperm after mating with a related male, than the following mating with an unrelated male (figure 1 and table 1*a*). Further investigating the relative role of genetic relatedness *per se* and MHC similarity, we found that models including similarity at MHC class I minor, and MHC class I minor together with genetic relatedness, were a much better explanation of the data than models including relatedness alone or including similarity at any of the other MHC loci or overall MHC similarity (table 1*a*; electronic supplementary material, table S1).

**Table 1.** Selection of models explaining variation in female sperm retention. (*a*) ‘Highest sperm number on eggs, all clutches’, including both trials with and without clutches with sperm, (*b*) probability of female sperm retention (i.e. ‘sperm absence’—whether females laid clutches with sperm or not) and (*c*) extent of female sperm retention (i.e. ‘highest sperm number on eggs’—excluding females that laid clutches without sperm) after (i) ‘controlled natural mating’ and (ii) ‘artificial insemination’. Models are ranked according to their AICc value and weight (*ω*), where lower AICc values and higher *ω* values imply a better goodness of fit of models. Accepted convention is that models that have a change in AICc compared with best-ranking model (*Δ*AICc) of less than 2 are equivalent, whereas models with *Δ*AICc > 2 are less supported (and therefore not presented here). ‘Null models’ only contain random effects. For comparisons, ‘null models’ and models with ‘relatedness’ are shown in the table even when *Δ*AICc > 2. The relative importance of variables occurring in the best supported models (**∑**AIC*ω*) is presented in the electronic supplementary materials, table S1. Terms initially included in the models were: relatedness, MHC similarity (one of MHC class I minor, MHC class I major, MHC class II minor, MHC class II major, MHC overall similarity), oviposition day (the day the egg was laid that had the highest sperm number, for (*a*,*c*)), ejaculate volume (for artificial insemination) and ejaculate sperm number (for controlled natural mating), together with female identity and male identity.

model	AICc	*Δ*AICc	*ω*
(*a*) highest sperm number on eggs, all clutches			
(i) controlled natural mating			
(1) MHC class I minor + relatedness	192.80	0	0.50
(2) MHC class I minor	193.63	0.82	0.33
(3) relatedness	249.05	56.25	0
(4) null model	308.39	115.59	0
(*b*) sperm absence			
(i) controlled natural mating			
(1) relatedness	75.71	0	0.56
(2) null model	76.73	1.02	0.33
(ii) artificial insemination			
(1) null model	34.20	0	0.63
(2) relatedness	36.67	2.47	0.18
(*c*) highest sperm number on eggs (only clutches with sperm)			
(i) controlled natural mating			
(1) MHC class I minor	125.95	0	0.41
(2) relatedness	131.73	5.77	0.02
(3) null model	133.67	7.72	0.01
(ii) artificial insemination			
(1) null model	96.58	0	0.45
(2) MHC class 1 minor	98.29	1.71	0.19
(3) relatedness	99.08	2.50	0.13

**Figure 2.**
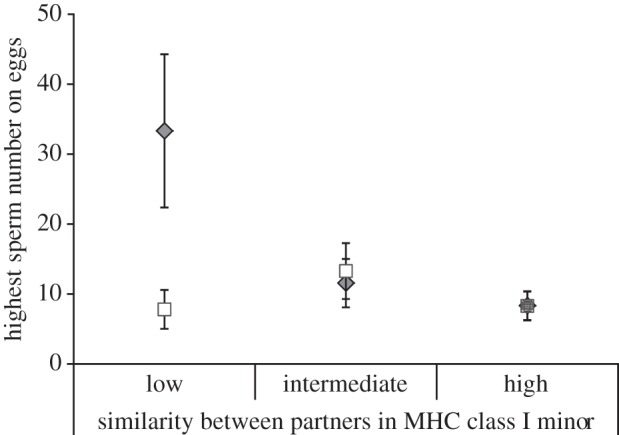
The relationship between extent of sperm retention (i.e. ‘highest sperm number on eggs’, only including clutches with sperm) for red junglefowl females that retained sperm (i.e. only including clutches with sperm; table 1*c*) after ‘controlled natural mating’ (filled diamonds) and ‘artificial insemination’ (open squares), and similarity between partners at the MHC class I minor locus (‘low’ similarity = 0, ‘intermediate’ similarity = 0.50–0.67, ‘high’ similarity = 1; ‘controlled natural mating’: *n*_clutches_ = 6, 20, 7, respectively; ‘artificial insemination’: *n*_clutches_ = 6, 17, 10, respectively). Data are represented as ±s.e.m. Controlled natural mating: *n*_clutches_ = 33, *n*_females_ = 29; artificial insemination: *n*_clutches_ = 33, *n*_females_ = 25.

We found no evidence that failure to transfer and/or store sperm was predicted by relatedness or MHC similarity (table 1*b*). In the more parsimonious analysis eliminating trials with such failures, we found that similarity at the MHC class I minor locus alone best explained the variation in the amount of sperm found on eggs (figure 2 and table 1*c*; electronic supplementary material, table S1), providing a substantially better explanation than any models that included relatedness or MHC similarity (table 1*c*; electronic supplementary material, table S1).

We found no evidence that males in this experiment biased sperm investment in favour of unrelated or MHC-dissimilar females (table 2*a*; the tendency towards an effect of MHC class II major locus is further weakened by correction for multiple testing).

**Table 2.** Parameters potentially affecting (*a*) ‘ejaculate sperm number’ (i.e. sperm numbers allocated by focal males to females) and (*b*) probability of ‘female ejaculate ejection’ by red junglefowl, during controlled natural mating. No variables remained significant after correction for multiple testing.

parameter	*χ*^2^	*p*
(*a*) ejaculate sperm number		
relatedness	1.57	0.21
MHC overall similarity	15.89	0.10
MHC class I minor	0.51	0.92
MHC class I major	0.67	0.88
MHC class II minor	1.69	0.64
MHC class II major	7.32	0.06
social familiarity	4.07	0.04
(*b*) female ejaculate ejection		
relatedness	1.67	0.20
MHC overall similarity	5.33	0.87
MHC class I minor	2.15	0.54
MHC class I major	2.75	0.43
MHC class II minor	2.21	0.53
MHC class II major	1.88	0.60
social familiarity	2.52	0.11
ejaculate sperm number	5.60	0.02

Female ejaculate ejection was not predicted by the genetic relatedness or MHC similarity between a female and a male (table 2*b*).

Following artificial insemination, we detected no effect of either MHC similarity or genetic relatedness on the probability that any sperm reached the eggs (table 1*b*). Similarly, models including MHC similarity or relatedness did not predict variation in the number of sperm found in clutches with sperm presence following artificial insemination any better than the null model (table 1*c*).

## 4. Discussion

In the red junglefowl, we found that variation in failure to transfer and/or store sperm was not predicted by relatedness or overall MHC similarity among partners following natural copulations. However, females did bias subsequent sperm use in response to genetic relatedness among partners, with more sperm reaching eggs after mating with males that were dissimilar at the MHC class I minor locus. This bias was not explained by male differential sperm allocation or female ejaculate ejection. Intriguingly, this bias was no longer detected following artificial insemination.

A bias in the number of sperm found on the PVL of eggs might, in principle, be owing to differential sperm allocation by males in response to relatedness [38] or MHC similarity [50]. However, in experimental mating trials, we found no evidence that males biased sperm investment in favour of unrelated or MHC-dissimilar females. This strongly suggests that our results are owing to an active bias in sperm selection driven by females and confirms earlier observations of cryptic female choice in this population [38]. One mechanism of cryptic female choice that is well documented in this species is differential ejaculate ejection through cloacal contractions immediately after mating [37,39]. This mechanism was suggested to underpin the detected female bias against sperm inseminated by related males found in a previous study [38]. However, we found no evidence that female ejaculate ejection was predicted by male–female genetic relatedness or MHC similarity. It is therefore likely that the observed cryptic female choice in favour of MHC-dissimilar males is owing to physiological processes governing the fate of spermatozoa beyond the female cloaca, for example through differential sperm retention in the female sperm storage tubules [66,67]. Such female-driven physiological processes might be triggered either by the recognition of the MHC similarity of an ejaculate within the female oviduct, or by the female perception of MHC similarity based on phenotype of the mating male.

In an attempt to reveal which cues trigger the MHC-dependent cryptic female choice observed, we performed an artificial insemination experiment in which female responses to male mating behaviour and phenotypic cues are entirely removed. If females require exposure to a male in order to bias sperm use in favour of MHC-dissimilar partners, artificial insemination should weaken or altogether eliminate the bias in female sperm use. Consistent with this prediction, we detected no bias in the number of sperm on the egg PVL following artificial insemination. These results indicate that the observed sperm bias towards sperm of MHC-dissimilar males in natural copulations was lost following artificial insemination.

The possibility that females cryptically bias sperm use to either avoid inbreeding or optimize offspring MHC diversity has attracted intense research interest [12,19,21]. However, results have been rather ambiguous; while some studies found evidence of cryptic female choice to avoid inbreeding [68,69], others have failed to find such effects [70–72]. Studies showing an effect of the MHC on postcopulatory prezygotic processes are scarce, and the mechanisms underlying the observed biases are unknown. In Arctic charr, *Salvelinus alpinus*, MHC-heterozygous males gained more fertilizations when in competition with MHC-homozygous males [26]. By contrast, in Atlantic salmon, *Salmo salar*, males gained more fertilizations when they were similar (rather than dissimilar) at MHC class I loci [28]. Cryptic female choice in externally fertilizing species like these is likely to be limited to sperm–egg interactions owing to a lack of internal interaction with the female. Studies of MHC-dependent effects in internal fertilizers are even scarcer. In mice, *Mus musculus*, Wedekind *et al.* [73] observed non-random production of blastocysts *in vitro*, biased towards MHC heterozygosity. Furthermore, MHC-heterozygous parents produced more heterozygous embryos than expected when infected with mouse hepatitis virus [74]. Selective sperm–egg interaction was suggested as a potential mechanism explaining these biases [73,74]. Importantly, in most studies it has been difficult to disentangle the possible independent effects of genome-wide relatedness and MHC similarity—two variables that are often closely correlated [32,33]. Therefore, another unresolved question is the extent to which such female choice functions solely as a means of inbreeding avoidance or if it is driven by the genetic benefits associated with MHC heterozygosity above and beyond avoiding the deleterious effects of inbreeding. Recent studies of red junglefowl and domestic breeds represent a typical case in point. Studies reported evidence of cryptic female choice against inbreeding in female red junglefowl [38] and cryptic female choice owing to genetic compatibility between different breeds of domestic fowl [63]. However, the role of MHC similarity in these responses was unknown despite recent demonstrations that MHC heterozygosity can affect sexual selection and survival in the red junglefowl [10,50]. The present results not only confirm previous evidence showing that cryptic female choice reduces the risk of inbreeding in this species [38], but also reveal that this is achieved by a more focused female postcopulatory bias in favour of dissimilarity at specific MHC loci.

Both natural and artificial insemination is known to trigger immune response in the female reproductive tract in domestic fowl [67], indicating a possible route through which the MHC may mediate sperm selection. However, our results show that the cryptic female responses to genetic similarity observed after natural copulations disappear after artificial insemination, possibly owing to a lack of male stimuli. Previous artificial insemination studies in birds have failed to find evidence of cryptic female choice in response to relatedness [72]. A possible explanation for this is that female selective barriers might need to be activated by the female's perception of male phenotypic cues, which are removed in artificial insemination. That male phenotypic cues are important in female reproductive decisions is well known [75,76]. Nevertheless, our study is the first to indicate that a direct link between the absence of precopulatory male cues and a loss of postcopulatory female discrimination might occur. This interpretation requires a degree of caution. Alternative explanations for the lack of cryptic female choice following artificial insemination include the possibility that semen samples collected from abdominal massage from the males might differ in some way from natural ejaculates (e.g. the absence of specific seminal factors), and thus prevent cryptic female choice. Similarly, it is possible that the process of artificial intromission of the ejaculate might bypass sperm barriers in the very first section of the female vagina. At present, these alternative hypotheses appear unlikely as neither is corroborated by what we currently know about the reproductive physiology of the fowl.

Surprisingly, the MHC effect observed in our study appears to be mediated by a single specific locus (out of the four MHC loci present in fowl). MHC variables were moderately to highly intercorrelated in our dataset, thus it is difficult to fully separate the effects of the independent loci. Nevertheless, we consistently find that similarity at a single MHC locus (class I minor locus) predicts a bias in female sperm use, but similarity at the other MHC loci (or across all pooled loci) does not. This finding may shed light on the functions of different MHC loci. In fowl, minor loci are expressed 10-fold less than major loci and are suggested to play a limited role in antigen presentation [44]. Moreover, it has been suggested that the MHC class I minor locus may have alternative, more specific functions than identifying pathogens [40,44]. Indeed, our results demonstrate that testing the effect of the MHC across pooled loci may miss the more complex processes occurring as a result of variation at individual MHC loci [77].

In conclusion, we demonstrate that in the red junglefowl, MHC-dissimilarity between partners, specifically at the MHC class I minor locus, explains a bias in female sperm use after copulation. Our results indicate that female-driven biases in sperm use are complex and may integrate responses both to relatedness *per se* and to MHC similarity. We suggest that future research should focus on exploring the exact cues and mechanisms of cryptic female choice, and the adaptive function of fertilization biases mediated by specific MHC loci.

## Data accessibility

Data deposited in the Dryad repository (dryad.org): http://dx.doi.org/10.5061/dryad.41311
